# SPATIAL MODELING OF RISK FACTORS FOR UNDER-FIVE PNEUMONIA IN ROKAN HILIR DISTRICT, INDONESIA

**DOI:** 10.21010/Ajidv19i2.3

**Published:** 2025-04-07

**Authors:** YUSDIANA Yusdiana, SUKENDI Sukendi, SIREGAR Yusni Ikhwan, AFANDI Dedi

**Affiliations:** 1 Doctor of Environmental Science, Postgraduate Program, Universitas Riau, Pekanbaru 28131, Indonesia

**Keywords:** Under-five pneumonia, Risk factors, Spatial model, Vulnerable areas, Indonesia

## Abstract

**Background:**

Under-five pneumonia remains a critical health issue in Indonesia. Identifying risk factors using spatial models is crucial for developing effective disease-prevention strategies. This study aimed to identify risk factors and create a spatial model for under-five pneumonia distribution based on regional vulnerability.

**Materials and Methods:**

This study used a mixed-method approach that integrated mathematical models and GIS to identify risk factors using generalized Poisson regression (GPR) and developed a GIS-based spatial model with inverse distance weighted (IDW) and natural break methods.

**Results:**

The GPR model revealed significant associations between under-five pneumonia and population density (β = 0.004, Z_-score_ = 6.118), rainfall (β = 0.002, Z_-score_ = 6.031), malnutrition (β = 1.786, Z_-score_ = 3.696), and health facilities (β = 0.073, Z_-score_ = 13.527). Protective factors included exclusive breastfeeding (β = -0.004, Z_-score_ = -2.874), healthy homes (β = -0.021, Z_-score_ = -9.532), and under-five health service coverage (β = -0.003, Z_-score_ = -2.225). Spatial modeling classified regions into high-risk (5 subdistricts), medium-risk (11 subdistricts), and low-risk (3 subdistricts).

**Conclusion:**

This study identified key risk factors and mapped spatial vulnerability for under-five pneumonia. Targeted, integrated interventions in high-risk areas are essential to reduce pneumonia incidence below 12 cases per 1,000 children under five by 2030, aligning with global health goals.

## Introduction

Pneumonia is an environmental disease that can cause serious health problems. Its incidence continues to increase significantly in several countries. Pneumonia is one of the leading causes of death in children under five years of age, surpassing other infectious diseases, including measles and AIDS (Hockenberry *et al.*, 2023). An estimated 1.2 million children under five years of age die from pneumonia each year (Anjaswanti *et al.*, 2022), equating to more than 2,200 deaths every day, including over 153,000 newborns, in 2018 (UNICEF, 2020). It is expected to remain the leading cause of death among children under five until 2040 (Wangdi *et al.*, 2021). Pneumonia is a significant cause of childhood morbidity and mortality, particularly in developing countries (Tintinalli *et al.*, 2016; Cillóniz *et al.*, 2021; Kusumadewi *et al.*, 2023).

More than half of the new cases of under-five pneumonia occur in five countries: India (44 million), China (18 million), Nigeria and Pakistan (7 million each), and Bangladesh and Indonesia (6 million each) (WHO, 2014). According to data from the World Health Organization, there were 25,481 under-five deaths due to pneumonia in Indonesia, ranking seventh among the countries with the highest pneumonia burden in the world (WHO, 2021). According to the 2018 Indonesian Basic Health Research Report, the prevalence of pneumonia among under-fives in Indonesia reached 2.1 percent, with the highest prevalence occurring in children aged 12-23 months (MoH, 2019).

Pneumonia is also the leading cause of death in children under 11 months of age (14.5%), and the second leading cause of death in children aged 12-59 months, with a proportion of 5.05% (MoH, 2021). Riau Province has a high rate of under-five pneumonia cases, with a prevalence of 1.46 percent. An estimated 18,241 under-fives in Riau Province suffered from pneumonia in 2019, with the Rokan Hilir District being one of the areas with a high prevalence of under-fives pneumonia, reaching 2.04 percent (DoH Riau, 2019). There were 1,954 cases of under-five pneumonia in the Rokan Hilir Regency, accounting for approximately 10.71% of the total cases in Riau Province in 2020 (DoH Riau, 2020). Pneumonia is an infectious disease caused by several agents including viruses, bacteria, and fungi. The most common causes in HIV-infected children are *Streptococcus pneumoniae, Haemophilus influenzae type b* (Hib), *respiratory syncytial virus*, and *Pneumocystis jiroveci* (Kusumadewi *et al.*, 2023).

Streptococcus infections can occur in endemic areas and in immunodeficient children. Therefore, authorities should consider local and regional epidemiological factors (Tintinalli *et al.*, 2016), which require analysis of risk factors and spatial distribution patterns.

The increase in under-five pneumonia cases in the Rokan Hilir Regency is related to many determining variables associated with features of airborne infections. Demographic traits, climatic variability, socioeconomic status, and the availability of health infrastructure are factors that require careful consideration. Furthermore, disregarding the patterns of disease spread further complicates efforts to address under-five pneumonia as an airborne illness. Understanding epidemiological principles, identifying factors, and comprehending the patterns of illness distribution are essential prerequisites for devising effective preventive and control methods. Examination of these risk factors has led to the identification of factors that can contribute to disease prevention and control. Knowing disease distribution patterns helps to identify high-risk locations, understand the environmental and socioeconomic variables leading to disease spread, and provide information essential for establishing targeted intervention programs.

Comprehensive efforts are required to address this concern, including gathering data on factors and distribution patterns associated with pneumonia epidemiology. Risk factor modeling and the use of GIS-based technologies can help identify previously unseen risk factors and distribution patterns. In the last decade, disease epidemiology using mathematical and spatial modeling has been widely used as a data analysis and visualization tool for structuring disease interventions (Robinson, 2000). The application of GIS helps recognize spatial differences, patterns, and risk factors of disease and improves health services (Jaber *et al.*, 2022). Spatial analyses have been widely applied to several infectious diseases (Openshaw, 1996; Kitron, 1998; Bergquist, 2001; Sithiprasasna *et al.*, 2004; FD *et al.*, 2023) and play an important role in disease surveillance and control (Albrecht, 2024).

The intricacy of risk factors and geographical distribution patterns of under-five pneumonia in the Rokan Hilir District warrant detailed scientific research. Unlike other airborne illnesses, such as TB, which has a national preventive program via directly observed treatment short course (DOTS), under-five pneumonia does not have a preventative program, particularly at the local level. Attention to the spread of under-five pneumonia is now only at the level of manually collecting case findings and short-term treatment of suspected cases. Therefore, information on the risk factors and distribution patterns of under-five pneumonia cases in Rokan Hilir District has never been researched or published. This study aimed to precisely identify and assess the risk factors that impact the growth of under-five pneumonia cases and their distribution patterns using mathematical and geographical modeling. The novelty of this study is the development of a method for prioritizing spatial interventions to prevent pneumonia in children under five years of age by effectively considering the vulnerability levels of different regions.

## Materials and Methods

### Study Area

**Figure 1:**
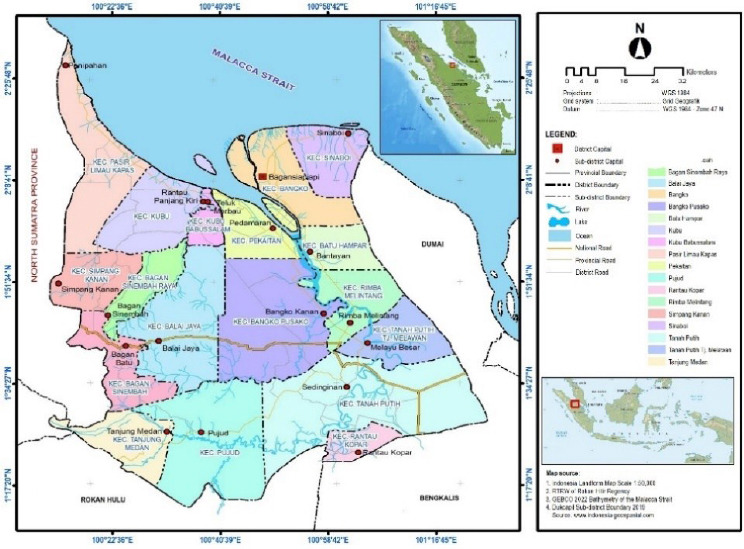
Study area location

The District of Rokan Hilir is situated at latitudes of 1° 14’ 0″ N to 2° 45’ 0″ N and longitudes of 100° 17 ’ 0″ E to 101° 21’ 0″ E (Figure 1). It encompasses an area of 8,881.59 km², bordered by the Strait of Malacca to the north, Dumai City to the east, Kampar Regency and Bengkalis Regency to the south, and Labuhan Batu Regency of North Sumatra Province to the west. Administratively, 18 sub-districts served as units of analysis, including Bagansiapiapi (capital), Tanah Putih, Pujud, and Tanah Putih Tj. Melawan, Rantau Kopar, Tanjung Medan, Bagan Sinembah, Simpang Kanan, Bagan Sinembah Raya, Balai Jaya, Kubu, Pasir Limau Kapas, Kubu Babussalam, Bangko, Sinaboi, Batu Hampar, Pekaitan, Rimba Melintang, and Bangko Pusako.

### Research design

This research employed a mixed-method design comprising an observational study and GIS-based spatial approach. Risk factors as independent variables included demographics (population density, under-five population), climate variability (temperature, humidity, rainfall, PM 2.5 µm), socioeconomic status (exclusive breastfeeding, Clean and Healthy Lifestyle-CHL, malnutrition, healthy homes, poor population), and health infrastructure (under-five health services, health workers, and health facilities). The dependent variable was the number of under-five pneumonia cases in Rokan Hilir District. This study used observational methods to examine under-five pneumonia cases, demographic data, socioeconomic status, and health infrastructure. Data were gathered by extracting variable information from several publications and documents published by authorized entities in 2021. Meanwhile, climatic variability data (air temperature, air humidity, rainfall, PM 2.5 µm concentration) were collected using a GIS-based spatial technique.

### Data collections

This study examined subdistrict-level aggregate data based on observations of denominator data linked to the results of all under-five pneumonia cases documented in the P_2_ISPA Report 2021 of the Rokan Hilir District Health Office. Data on demographic factors and health infrastructure were acquired from observations of data available on the Rokan Hilir Regency Statistics Agency (BPS) website *(https://rohilkab.bps.go.id/)*, specifically from the “Rokan Hilir District in Figures 2021”. Demographic data were screened for population density and under-five populations by sub-district. Health infrastructure factor data were screened based on the number of health workers, including doctors, nurses, midwives, pharmacists, and nutritionists, and the number of health facilities, including hospitals, polyclinics, puskesmas, pustu, and pharmacies. The percentage availability of health workers and health facilities is the ratio of the number of health professionals and health facilities to the total population.

Data on socioeconomic factors were obtained from the Rokan Hilir District Health Office report in the “Rokan Hilir District Health Profile” document of 2021. Screening of social factor data was carried out on the percentage of exclusive breastfeeding achievement and CHL by sub-district. Screening of economic factor data was conducted on the percentage of malnutrition among children under five years of age, healthy homes, and poor people in 2021.

Climate variability data (air temperature, air humidity, rainfall, PM 2.5 µm concentration) were collected using a GIS-based spatial method through satellite image data gathering and spatial data with interpolation and reclassification procedures. Temperature and humidity data were collected from the Modern-era Retrospective Analysis for Research and Applications version 2 (MERRA-2) provided by NASA USA *(https://gmao.gsfc.nasa.gov)* with a resolution of 0.5° × 0.5°. Average rainfall data were gathered from the Climate Hazards Group InfraRed Precipitation with Station (CHIRPS) worldwide using the US Geological Survey (USGS) via a portal *(http://iridl.ldeo.columbia.edu/SOURCES/UCSB/CHIRPS/)* with a resolution of 0.05° × 0.05°. PM 2.5 µm concentration data were collected from the monthly MERRA-2 data in the DMS Surface Mass Concentration (ENSEMBLE) M2TMNXAER_5.12.4 data archive published by NASA-USA *(https://giovanni.gsfc.nasa.gov/)* at a resolution of 0.5° × 0.625°.

**Figure 2:**
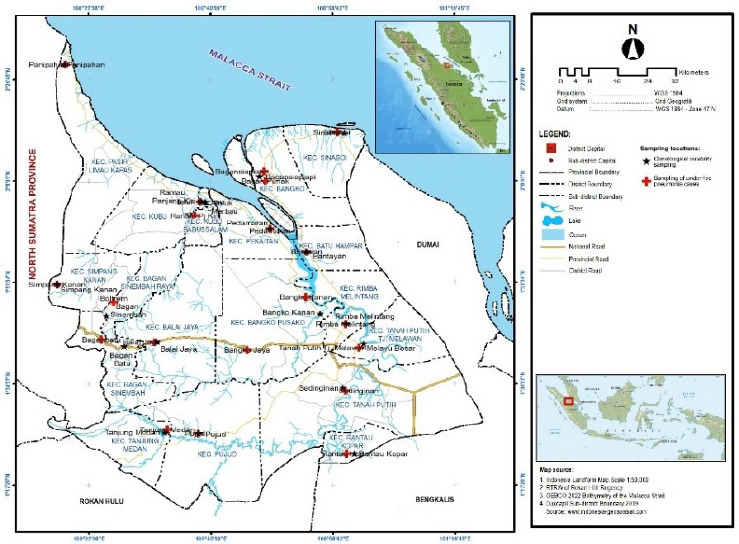
Sampling of under-five pneumonia cases and climate variability

Observations of under-five pneumonia medical record data were undertaken at 18 governmental health institutions in 2021, followed by the collection of climate variability data at 18 geographical observation locations as representatives of climatic conditions in the study region in 2021 (Figure 2). Table 1 presents the study variables, definitions, and sources.

**Table 1:** Research variables and their definitions and sources

Variable	Definition	Sources
**Under-five pneumonia**		
(1)Number of under-fives with pneumonia	Number of under-five pneumonia cases recorded in medical records at government health facilities.	(1) Observation by the author of medical record data of children under-five with pneumonia in government health facilities in the P2ISPA report of the Rokan Hilir District Health Office from 2015-2021 (DoH Rohil, 2022).
**Demographics**		
(2)Population density	Average population per km^2^ of subdistrict area (people/km^2^).	(2,3) Data observation by the author of the *online document* of Rokan Hilir Regency in Figures in 2021 (*https://rohilkab.bps.go.id/*) (CSA Rohil, 2021).
(3)Under-five population	Average under-five population is 10% of the total population by sub-district.
**Climatology**		
(4)Air temperature	Average air temperature (ºC) by sub-district measured 2 m above the earth’s surface.	(4, 5) Calculations made by the author through data interpolation analysis from MERRA-2 NASA-USA with a resolution of 0.5° x 0.5° in 2021 *(https://gmao.gsfc.nasa.gov)* using GIS.
(5)Air humidity	Average air humidity (%) by subdistrict.	
(6)Rainfall	Average rainfall per year (mm) by subdistrict.	(6) Calculation by the author through data interpolation analysis from the global Climate Hazards Group InfraRed Precipitation with Station (CHIRPS) with a resolution of 0.05o x 0.05o in 2021 *(http://iridl.ldeo.columbia.edu/ SOURCES/.UCSB/.CHIRPS/)* using GIS.
(7)PM 2.5 concentration	Average monthly PM 2.5 concentration (μm) in ambient air by subdistrict.	(7) Calculation by the author through interpolation analysis of DMS Surface Mass Concentration (ENSEMBLE) M2TMNXAER_5.12.4, MERRA-2 NASA-USA data with a resolution of 0.5° x 0.625° in 2021 *(https://giovanni.gsfc.nasa.gov/.)* using GIS.
**Social**		
(8)Exclusive breastfeeding	Number of children under-five 0-6 months who are solely breastfed (without supplementary food and drink except medication and vitamins) divided by the total number of infants under-five 0-6 months by sub-district multiplied by 100%.	(8, 9) Observation by the author of the Rokan Hilir Regency Health Profile document by the Rokan Hilir Regency Health Office in 2021 (DoH Rohil, 2021).
(9)CHL	Number of households that match the 10 requirements of CHL (according to MoH No. 2269/MENKES/PER/XI/2011) divided by the total number of families per sub-district multiplied by 100%.
**Economics**		
(10)Malnutrition	Population of under-fives who are extremely malnutrition, defined by weight, height, or upper arm circumference that is considerably below the normal level for their age (according to MoH No. 2 Year 2020), divided by the total population of under-fives per sub-district multiplied by 100%.	(10, 11) Observation by the author of the Rokan Hilir Regency Health Profile document by the Rokan Hilir Regency Health Office in 2021 (DoH Rohil, 2021). (12) Observation by the author of the *online document* of Rokan Hilir Regency in Figures in 2021 (*https://rohilkab.bps.go.id/*).
(11)Healthy home	Number of healthy homes that match the 7 requirements of a healthy dwelling (according to MoH No. 1077/MENKES/PER/V/2011) divided by the total number of houses per sub-district multiplied by 100%.	
(12)Poverty	Number of persons earning less than the average minimal per capita spending per month to fulfil basic necessities divided by the entire population by sub-district multiplied by 100%.
**Healthcare infrastructure**		
(13)Under-five health services	Number of children under-five who received basic health services including health checks, immunizations, vitamin and medicine administration, and nutrition consultation (according to MoH No. 25 of 2014) at government-owned health service facilities divided by the total number of children under-five by sub-district multiplied by 100%.	(13, 14, 15) Observation by the author of the *online document* of Rokan Hilir Regency in Figures in 2021 (*https://rohilkab.bps.go.id/*).
(14)Health worker availability	Number of health personnel comprising doctors, nurses, midwives, pharmacists, and nutritionists.	
(15)Health facility availability	Number of health facilities comprising hospitals, polyclinics, health centers, and pustu.
**Spatial distribution**		
(16)Risk factors spatial distribution	Classification of the geographical distribution of risk factors of under-five pneumonia cases by subdistricts with high, medium, and low categories.	(16) Interpolation by the authors through GIS using the IDW method (Azpurua and Dos Ramos, 2010) and Natural Breaks classification (Dent, 1999; Hibatullah, 2016; Slocum *et al.,* 2022; Harahap *et al.,* 2023)
(17)Area vulnerability level	Classification of the geographical distribution of the vulnerability of under-five pneumonia cases by subdistrict based on the score results of the risk factors with categories of high, medium, and low-risk regions.	(17) Interpretation by the author through GIS using overlay techniques and Natural Breaks classification (Dent, 1999; Hibatullah, 2016; Slocum *et al.,* 2022).

### Data analysis

Statistical data processing using R Studio rev 2022.07.1+554 software and ArcGIS Desktop 10.8 rev.10.7.0.10450 for GIS-based spatial analysis. Descriptive analysis was used to provide an overview of the data distribution for each study variable. The links between demographic variables; climatic variability; social, economic, and health infrastructure; and under-five pneumonia cases were studied using the GPR model. The GPR model is a nonlinear regression model used for count data in which the response variable follows a Poisson distribution (Utama and Hajarisman, 2021). Poisson distribution is the distribution of events that rely on a specific period or specific region using data in the form of discrete variables. The processes of developing the GPR model include: 1). A multicollinearity test was conducted using the VIF value, with the equation 
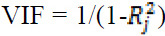
 dan *tol* = 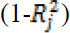 (R = correlation coefficient of independent variables/predictors; tol = tolerance of independent variables/predictors). The risk factor is declared free from multicollinearity if the VIF value is <10; if the VIF>10, the variable is removed from the model using the backward elimination method (Achmad *et al.*, 2022); 2) Classify the response (dependent) variable on a Poisson distributed discrete random variable with parameter β using the probability function equation *f*(*y,μ*) = 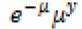/y_i_; y = 0,1,2 (μ = mean value and variance y>0); 3) Construct a Poisson regression model with the equation 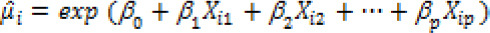 (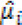 = average under-five pneumonia cases in a given time interval, β = constant, Xi = risk factor); 4) Testing the effect of the risk factor (x) on the response variable (y) with the equation *Z-_score_* = 
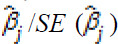
 for partial effect and the equation 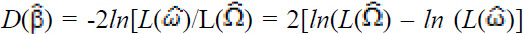 for simultaneous effect, provided that if the value of |*Z-_score_*| > *Z_α_*/2 with α = 0.05, then the risk factor (X) has a partially significant impact on the response variable (Y), if the value of 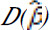 *> χ^2^_(p,α)_* means that there is at least one risk factor (X) that has a significant effect on the response variable (Y). Spatial data analysis was performed using the GIS approach through interpolation and reclassification techniques.

The spatial distribution of risk factors and the distribution of under-five pneumonia cases were interpolated using the IDW method with the equation 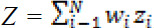, (*Z_i_* = the height value of data from number of N points, wi = weight value, x,y = coordinates of interpolation points, i = coordinates for each point distribution) (Azpurua and Dos Ramos, 2010). Spatial distribution classification uses the natural breaks method (Jenk optimization) based on EHDP (Dent, 1999; Hibatullah, 2016; Slocum *et al.*, 2022; Harahap *et al.*, 2023), which divides groups based on the value of the standard deviation of the least squares as the optimum regional divider (Ekawati *et al.*, 2021; Kassam *et al.*, 2021). Furthermore, data from the reclassification of natural breaks were digitized in the form of polygons based on sub-district administrative borders with important factors and the number of under-five pneumonia cases as characteristics. The vulnerability level of the region was evaluated into high, medium, and low-risk zones using a scoring approach for the determining variables, employing a Likert scale with the following parameters: low = 1, medium = 2, and high = 3 (Muriithi, 2023). The total risk score was calculated by summing the individual values for each determining factor. Furthermore, the locations were categorized based on the value of the natural break range, using an overlay approach.

## Results

### Risk factors of under-five pneumonia

According to the demographic variables, the average population density of Rokan Hilir District in 2021 is approximately 70 people/km², with substantial variance among the sub-districts. The highest population density was observed in the Bangko subdistrict (168 people/km²), whereas the lowest was observed in the Rantau Kopar subdistrict (29 people/km²).

The drivers of climatic variability suggest an average air temperature of roughly 26.52°C with large fluctuations across sub-districts, and the maximum air humidity is in the Bagan Sinembah sub-district (91.12%). Rainfall averaged approximately 3,127 mm/year, with the highest fluctuation occurring in the Simpang Kanan sub-district (3,470 mm/year). PM 2.5 µm values were approximately 11.68 µg/m³, with the highest in the Batu Hampar sub-district (13.27 µg/m³).

Social factors revealed a low rate of exclusive breastfeeding (41.13%), with only a few subdistricts having values exceeding 50%. The Tanjung Medan sub-district has the highest proportion of clean and healthy homes (100%), whereas the Sinaboi sub-district has the lowest proportion (17.22%). Regarding economic factors, the proportion of families with healthy housing status is relatively high (71.11%), although poverty factors are still substantial, with an average percentage of approximately 46.21%.

Health infrastructure factors demonstrate that the coverage of under-five healthcare varies among subdistricts, with certain subdistricts having an attainment rate exceeding 100%. The number of health professionals tends to be inconsistent among sub-districts, with a ratio of 2.9 health workers per 1,000 inhabitants. The distribution of health facilities of government-owned health institutions is reasonably equitable, with hospitals, polyclinics, and pharmacies still concentrated in metropolitan areas and certain sub-districts with restricted access. A description of the risk factors for under-five pneumonia cases in Rokan Hilir District is presented in Table 2.

**Table 2:** Descriptive statistics on risk factors of under-five pneumonia cases in Rokan Hilir District

Risk factors	Unit	Minimum	Maximum	Mean	Std. Dev	Variance
**Demographic**						
Population density	people/km^2^	29	168	69.78	40.25	1,620.06
**Climatology**						
Air temperature	^0^C	25.76	28.27	26.52	0.71	0.51
Air humidity	%	78.31	91.12	87.71	3.92	15.36
Rainfall	mm	2.917	3.470	3,127.44	156.56	24,509.91
PM 2.5 concentration	µm	10.31	13.27	11.68	1.20	1.45
**Social**						
Exclusive breastfeeding	%	1.66	79.81	41.13	20.78	431.63
CHL	%	17.22	100.00	53.65	22.90	524.62
**Economic**						
Malnutrition	%	0.00	0.31	0.056	0.081	0.007
Healthy home	%	47.00	93.00	71.11	15.28	233.63
Poverty	%	19.68	70.39	46.21	12.79	163.71
**Health infrastructure**						
Under-five health services	%	23.67	113.96	61.86	25.34	642.12
Health worker availability	person	34	468	104.94	107.78	1,1614.06
Health facility availability	unit	3	23	10.39	6.26	39.19

The correlation between the risk factor variables and under-five pneumonia cases in the Rokan Hilir Regency was investigated using a Poisson regression model. One of the requirements that must be met in the preparation of the Poisson regression model is the fulfilment of multicollinearity requirements by examining the Varian Inflation Factor (VIF) value of each risk factor with the provisions that if the VIF value <10, then there is no multicollinearity case; otherwise, if the VIF value >10, then there is a multicollinearity case. The multicollinearity test results revealed that the risk factors of air temperature, air humidity, and the number of health workers had a VIF>10, which suggests that there is a case of multicollinearity in the three variables; thus, they must be excluded from the model.

The risk factors of population density, rainfall, PM 2.5 µm concentration, exclusive breastfeeding, CHL, malnutrition in children under five years, healthy homes, poor population, coverage of health services for children under five years, number of health workers, and number of health facilities were continued as candidate risk factors to compose the model. The autocorrelation checks between candidate risk factors using the residual run test was 1.00 (>0.05), which shows that there is no autocorrelation between risk factors and fits the criteria for the Poisson regression model of under-five pneumonia cases. The model of the correlation between the risk factors and under-five pneumonia cases is shown in Table 3.

**Table 3:** Correlation risk factors model with under-five pneumonia cases in Rokan Hilir District

Variable	Estimation	Std. Errors	Z-scores
β0	8.306	1.0630	7.814
Population density	0.004	0.0007	6.118
Rainfall	0.002	0.0003	6.031
PM 2.5 concentration	0.077	0.0481	1.598
Exclusive breastfeeding	-0.004	0.0015	-2.874
Malnutrition	1.786	0.4834	3.696
Under-five health services	-0.003	0.0015	-2.225
CHL	-0.001	0.0016	-0.472
Healthy home	-0.021	0.0022	-9.532
Poverty	0.001	0.0033	0.439
Health facility availability	0.073	0.0054	13.527

Devians:118.56			
AIC: 253.97			
*Ztable*(0.05):1.65			
*χ^2^*(0.1:10):15.987			
Significance *α* = 0.1			

**Figure 3:**
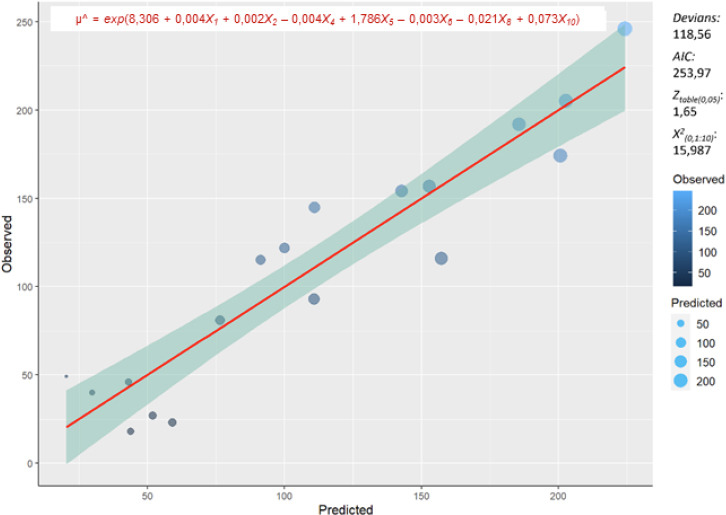
Model of correlation risk factors for under-five pneumonia cases in the Rokan Hilir District.

Testing the risk factors of the Poisson regression model yielded a deviance value of 118.56 (>χ^2^_(0.1:10)_ = 15.987), which suggests that all factors simultaneously impact the distribution of under-five pneumonia cases. A graphical representation of the correlation model between risk factors and under-five pneumonia cases in Rokan Hilir District is presented in Figure 3.

Partial testing of the risk factors of the model showed that the factors of population density, rainfall, exclusive breastfeeding, malnutrition in toddlers, toddler service coverage, healthy homes, and number of health facilities had a Z_-score_ > Z_table_ (1.65), which means that they had a significant partial effect on under-five pneumonia cases. Meanwhile, the factors of PM 2.5 µm concentration, CHL, and poor population had Z_-scores_ > Z_table_ (1.65), which suggests that they do not have a significant partial influence on under-five pneumonia. These data imply that population density, rainfall, exclusive breastfeeding, malnutrition, under-five service coverage, healthy houses, and several health facilities are the main predictors of under-five pneumonia cases in Rokan Hilir District.

The Poisson regression model equation is 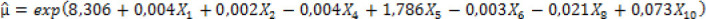, where the most sensitive risk variable for increasing the frequency of under-five pneumonia cases in the Rokan Hilir District is malnutrition. Each one percent rise in under-five malnutrition (*exp X_5_* = 1.786) resulted in an average increase of six occurrences of under-five pneumonia. A one percent increase in population density (*exp* *X_1_* = 0.004) and rainfall (*exp X_2_* = 0.002) resulted in an average increase of one incidence in under-five pneumonia cases. Meanwhile, a one percent increase in exclusive breastfeeding (*exp X_4_* = -0.004), under-five healthcare coverage (*exp X_6_* = -0.003), and healthy houses (*exp X_8_* = -0.021) would lower the average number of under-five pneumonia cases. Similarly, a one percent increase in the number of health facilities (*exp X_10_* = 0.073) increased the average number of under-five pneumonia cases. Model estimates of increases or decreases in under-five pneumonia cases based on major drivers assumed that other factors did not change over the projected period (constant).

### Spatial model of under-five pneumonia

**Figure 4:**
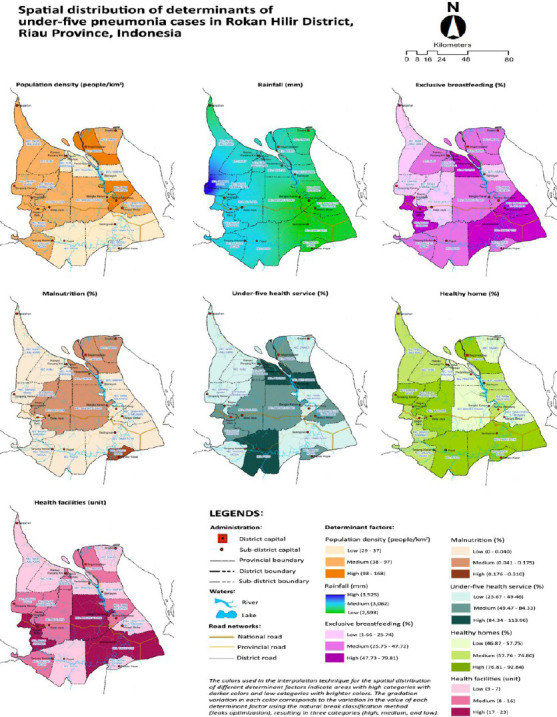
Spatial distribution of risk factors of under-five pneumonia in Rokan Hilir District, Riau Province, Indonesia

The distribution pattern of under-five pneumonia cases in the Rokan Hilir District differs among subdistricts. This condition is affected by the geographical distribution of important predictive factors that directly affect the variations in the number of under-five pneumonia cases as response variables. Based on the categorization of natural breaks, the distribution of the risk factors of under-five pneumonia cases across sub-districts was categorized into three: high, medium, and low. The spatial distribution of the risk factors for under-five pneumonia is shown in Figure 4.

The geographical distribution of demographic factors was represented by the population density variable, which was classified into three groups: high (98–198 people/km²), medium (38–97 people/km²), and low (29–37 people/km²). Sub-districts with low density include Kubu Babussalam, Pekaitan, Batu Hampar, Pujud, Tanah Putih, and Rantau Kopar. The medium category is located in the sub-districts of Pasir Limau Kapas, Kubu, Sinaboi, Simpang Kanan, Bagan Sinembah Raya, Balai Jaya, Bangko Pusako, Bagan Sinembah, Tanah Putih Tj. Melawan, and Tanjung Medan. High density is centred in the Bangko and Rimba Melintang subdistricts.

The decisive element of climatic variability is the rainfall variable in the Rokan Hilir Regency, which ranges from 2,598 to 3,525 mm. The sub-districts with the highest rainfall (3,525 mm) include the Simpang Kanan sub-district and a tiny section of the Pasir Limau Kapas sub-district, whereas the lowest rainfall (2,598 mm) is located in Rantau Kopar, Tanah Putih, and Tanah Putih Tj. Melawan, Rimba Melintang, and Bangko Pusako. The remaining 11 sub-districts received moderate rainfall (2,598–3,062 mm).

The geographical distribution of socioeconomic factors was represented by the exclusive breastfeeding variable, which was classified into three groups: high (47.73–79.81%), medium (25.75–47.72%), and low (1.66–25.74%). Sub-districts with a high percentage of exclusive breastfeeding include Pekaitan, Bagan Sinembah, Tanah Putih, and Tanah Putih Tj. Melawan, medium categories include Bangko, Sinaboi, Kubu, Kubu Babussalam, Bangko Pusako, Rimba Melintang, Tanjung Medan, Pujud, and Rantau Kopar, while low categories are found in Pasir Limau Kapas, Simpang Kanan, Bagan Sinembah Raya, Balai Jaya, and Batu Hampar. Economic factors were reflected in the malnutrition and healthy house variables.

The spatial distribution of malnutrition in the high category (0.176–0.310%) is in the Rantau Kopar sub-district, the medium category (0.041–0.175%) in Bagan Sinembah Raya, Balai Jaya, Bangko Pusako, Pekaitan, Bangko, and Sinaboi, and the low category (0.000–0.040%) in Bagan Sinembah, Tanah Putih, Tanah Putih Tj. Melawan, Kubu, Kubu Babussalam, Rimba Melintang, Tanjung Medan, Pujud, Pasir Limau Kapas, Simpang Kanan, and Batu Hampar. While the spatial distribution of healthy houses in the high category (76.81–92.84%) is concentrated in Bangko, Simpang Kanan, Bagan Sinembah Raya, Balai Jaya, Pujud, Tanah Putih, and Rantau Kopar sub-districts, the medium category (57.76–76.80%) in Pasir Limau Kapas, Kubu, Bagan Sinembah, Tanjung Medan, Batu Hampar, and Rimba Melintang, and the low category (46.87–57.75%) in Sinaboi, Kubu Babussalam, Pekaitan, Bangko Pusako, and Tanah Putih Tj. Melawan.

The factors of health infrastructure are represented by the variables of service coverage for children under five and number of health facilities. The spatial distribution of toddler health service coverage in the high category (84.34–113.96%) is found in Pekaitan, Batu Hampar, and Pujud sub-districts, the medium category (49.47–84.33%) in Bangko, Kubu Babussalam, Balai Jaya, Bagan Sinembah, Bangko Pusako, Tanah Putih Tj. Melawan, and Rantau Kopar, and the low category (23.67–49.46%) in Pasir Limau Kapas, Kubu, Simpang Kanan, Bagan Sinembah Raya, Sinaboi, Rimba Melintang, Tanjung Medan, and Tanah Putih. Meanwhile, the spatial distribution of the number of health facilities in the high category (17–23 units) is centered in Bagan Sinembah, Bangko Pusako, and Tanah Putih sub-districts, the medium category (8–16 units) in Bangko, Simpang Kanan, Bagan Sinembah Raya, Kubu Babussalam, Balai Jaya, Rimba Melintang, and Tanjung Medan sub-districts, and the low category (3–7 units) in Pasir Limau Kapas, Kubu, Pekaitan, Sinaboi, Batu Hampar, Pujud, Tanah Putih Tj. Melawan, and Rantau Kopar.

**Figure 5:**
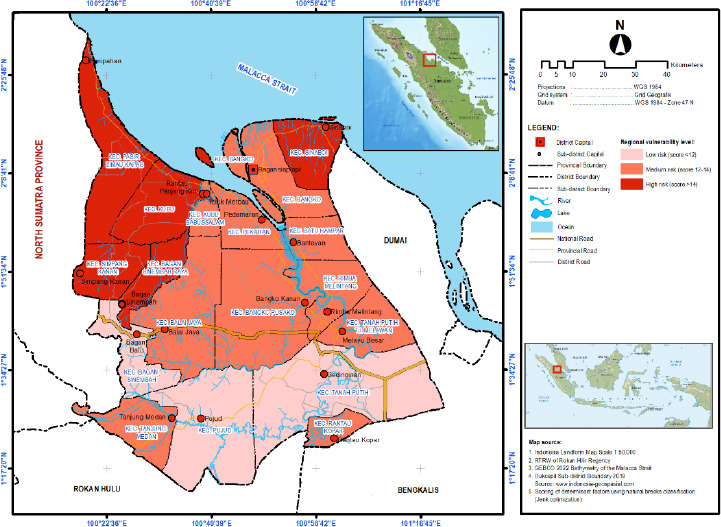
Spatial distribution map of the under-five pneumonia risk levels at the sub-district level based on scoring and overlaying risk factors

The geographical distribution map of the risk level of pneumonia in children under five years of age at the subdistrict level, based on scoring and overlay of the determining factors, is presented in Figure 5. The risk level of under-five pneumonia in Rokan Hilir District was determined by scoring the major variables and categorized using the Natural Breaks (Jenks Optimization) approach, resulting in three categories: high, medium, and low risk. High-risk regions (scoring >14) included the sub-districts of Pasir Limau Kapas, Simpang Kanan, Kubu, Bagan Sinembah Raya, and Sinaboi. Medium risk regions (scoring 12-14) include the sub-districts of Kubu Babussalam, Balai Jaya, Tanjung Medan, Pekaitan, Bangko Pusako, Bangko, Batu Hampar, Rimba Melintang, Tanah Putih Tj. Melawan, Tanjung Medan, and Rantau Kopar. Meanwhile, low-risk zones (score <12) comprise Tanah Putih, Pujud, and Bagan Sinembah sub-districts.

## Discussions

Population density in a region is a demographic parameter that strongly affects the rate of transmission of under-five pneumonia cases. Denser populations tend to have more intensive and frequent human contact, which increases the transmission of infectious pathogens to several airborne illnesses (Jin *et al.*, 2022). The Poisson regression model in the Rokan Hilir district indicated that somewhat similar dynamics in one incidence of under-five pneumonia would rise with every 0.70 person/km² increase in the population density. The same was also reported, noting that an increase in population density led to a rise in the number of under-five pneumonia cases in Java (Renika, 2021). A substantial association between population density and under-five pneumonia cases was also identified in Surabaya City, where every one percent rise in population density increased two instances of under-five pneumonia (Maghfiroh *et al.*, 2016). Population density has also increased five cases of pneumonia in Central Java Province (Utami and Yanti, 2021). A one-unit increase in Indonesia’s population density leads to one in five fatalities from pneumonia (Savitri *et al.*, 2022). In high-density areas, children under-five years of age are more commonly exposed to sources of illness via direct contact with diseased persons or through contaminated air. The spatial distribution of under-five pneumonia cases in the Rokan Hilir Regency is concentrated in urban areas with a relatively high population density, which tends to increase slums, hygiene problems, and malnutrition, thereby accelerating the spread of under-five pneumonia (Walker *et al.*, 2013). Spatially and temporally, an increase in under-five pneumonia cases is usually related to increased population density in urban regions (Sutriana *et al.*, 2021). Some strategic steps that can be taken to anticipate demographic factors include improving the facilities, quality, and accessibility of health services in densely populated areas; improving the quality of housing in urban areas to comply with sanitation and health standards to reduce the risk of spreading under-five pneumonia; increasing de-densification efforts through good spatial planning and construction of liveable housing in peripheral areas to reduce density in the city center; and local transmigration by relocating part of the population from dense areas to areas with lower populations, followed by adequate infrastructure development in the new areas. Based on spatial analysis, the sub-districts of Rantau Kopar, Batu Hampar, Pekaitan, Pujud, Tanah Putih, and Kubu Babussalam need to be prioritized for local transmigration and de-densification of affordable housing to relieve pressure in densely populated sub-districts. A fairer distribution of people via local transmigration initiatives is a successful strategy to considerably lower the transmission rate of airborne illnesses, such as tuberculosis (TB), in the Rokan Hilir Regency (FD *et al.*, 2023) .

Rainfall was the lead climatic variable that substantially affected the spread of pneumonia in Rokan Hilir District, in addition to other variables such as air temperature, air humidity, and PM2.5 µm concentration. This finding suggests that air temperature, air humidity, and PM2.5 µm concentration are not direct determinants of under-five pneumonia in the Rokan Hilir District. Jati and Ginandjar (2017) indicated that air temperature and humidity were not significantly associated with pneumonia despite their possible influence on acute respiratory infection (ARI) in girls. Similarly, the concentration of PM2.5 µm assessed in ambient air did not indicate a significant connection with an increase in under-five pneumonia infections, particularly in low-income and middle-income countries (Adaji *et al.*, 2019). The Poisson regression model in the Rokan Hilir area indicated an increase of one under five pneumonia cases for every 31.27 mm increase in rainfall. The mechanism of increased rainfall in the spread of under-five pneumonia is via an increase in the humidity of the microclimate within the home, which influences indoor air quality. This situation produces an optimal setting for the development and spread of harmful bacteria that cause respiratory tract infections in children under five years of age. In addition, stagnant water from excessive rain owing to inadequate household sanitation encourages the formation of disease vectors and enhances the spread of diseases via direct and indirect contact. The aggregation of these variables explains why the geographical distribution of high rainfall categories coincides with an increase in under-five pneumonia cases at the subdistrict level. Although the distribution of high rainfall improves macro-air quality and lowers particle concentrations, it causes the growth of indoor microorganisms to increase, thus affecting the incidence of under-five pneumonia, especially during the transitional season in tropical countries (Shetty and Shetty, 2009). Improving sanitation and drainage infrastructure to minimize waterlogging and decrease disease vector habitats are prioritized to prevent the development of pneumonia under five years of age due to excessive rain. Socialization and counseling on home health norms in susceptible locations should be addressed. Ensuring proper house ventilation and cleanliness will lower indoor humidity, thereby minimizing the incidence of under-five pneumonia despite the increasing rainfall.

Exclusive breastfeeding is a social factor that strongly impacts the growth of under-five pneumonia cases in Rokan Hilir District. Exclusive breastfeeding refers to providing breast milk as the sole source of nutrition for newborns, without the inclusion of supplemental fluids, solid foods, medications, or vitamins during the first six months of an infant’s life. Exclusive breastfeeding was a balancing factor in the Poisson regression model related to the spread of under-five pneumonia cases, where an increase in the percentage of exclusive breastfeeding tended to decrease in five pneumonia cases, whereas a decrease in exclusive breastfeeding increased in five pneumonia cases. The Poisson regression model revealed an average increase of one new under-five pneumonia case attributable to a drop in the proportion of exclusive breastfeeding of 0.41%. Rahmitri and Winahju (2016) reported a reduction in the average number of under-five pneumonia cases in Padang City when exclusive breastfeeding rose by one percent. The incidence of severe pneumonia in Bone Bolango District and Gorontalo City has dropped by 0.1 times, with a one percent increase in exclusive breastfeeding (Achmad *et al.*, 2022). A reduction in 165 cases of under-five pneumonia in Bali Province occurred because of an increase in the rate of exclusive breastfeeding (Dewi *et al.*, 2020). Toddlers with a history of not being exclusively breastfed had 7.4 times increased risk of pneumonia compared with toddlers with a history of exclusive nursing (Fikri, 2016). Exclusive breastfeeding strives to me *et al* the nutritional demands of newborns in the first six-month period since breast milk includes complete and sufficient nutrients for child growth and development and provides protection against many infectious illnesses. Optimal nutrition in breast milk creates antibodies that are crucial for the infant’s immune system; therefore, a low proportion of exclusive breastfeeding increases the vulnerability of under-fives to pneumonia (Lugonja *et al.*, 2024). Spatially, locations with poor exclusive breastfeeding rates are concentrated in vulnerable regions with insufficient under-five health facilities and services, especially Pasir Limau Kapas, Simpang Kanan, Sinaboi, Kubu, and Bagan Sinembah Raya sub-districts. Therefore, various strategic efforts need to be made, including intensive education and counseling programs on the importance of exclusive breastfeeding with a focus on mothers in vulnerable communities; provision of adequate support for breastfeeding mothers, including lactation counseling and support from trained health workers; public awareness campaigns involving community leaders and local media to change perceptions and habits about breastfeeding; and increasing the number of under-five health facilities and services that ensure a friendly environment for breastfeeding mothers, including comfortable breastfeeding rooms in public places and workplaces. Government policies that encourage appropriate maternity leave and protect nursing mothers’ rights in the workplace should be implemented to ensure that mothers have sufficient time and space to breastfeed their infants.

A clean and healthy lifestyle (CHL) was not significantly associated with the increase in under-five pneumonia cases in the Rokan Hilir District. Although numerous studies have explored the substantial link between CHL and under-five pneumonia, the findings of the Poisson regression analysis in this research revealed that CHL is not a direct driver of the increase in under-five pneumonia cases in the Rokan Hilir District. CHL enhances general health and minimizes the risk of infection by hand washing and by maintaining environmental hygiene (Samrah *et al.*, 2021). However, without particular treatments, such as vaccination against pneumonia-causing microorganisms or improving the nutritional condition of under-fives, CHL practices are not adequate to directly prevent or decrease the prevalence of under-fives pneumonia. Variations in the application of CHL at the home level in this study were not adequate to outweigh the protective influence of another social factor, exclusive breastfeeding. Although CHL was not a factor that directly influenced under-five pneumonia cases in this study, the implementation of CHL at the household level remains a fundamental foundation in efforts to enhance public health status for the prevention of different infectious illnesses in Indonesia (Kusuma, 2021).

Economic factors, including under-five malnutrition, percentage of healthy dwellings, and poverty rate, had varied impacts on the growth of under-five pneumonia cases in the Rokan Hilir District. The variables of under-five malnutrition and the proportion of healthy houses had a substantial effect on the increase in under-five pneumonia cases; however, the poverty variable showed no direct relationship. The Poisson regression model in the Rokan Hilir District showed an increase of six under-five pneumonia cases for every increase of 36 underweight children. Meanwhile, a 0.71% increase in the proportion of healthy households lowered the incidence of under-five pneumonia cases. These data imply that malnutrition is an essential predictor of the increase in under-five pneumonia cases, particularly in low-income countries (Imran and Imran, 2020). Lack of dietary intake impairs the immune system and respiratory muscles, limiting the process of clearing secretions in the respiratory tract of under-fives, rendering them prone to pneumonia (Alamneh and Adane, 2020). The significant relationship between malnutrition and an increase in under-five pneumonia cases is reinforced by the prevalence of stunting in the Rokan Hilir Regency in 2021, which has reached 29.7%, well above the average prevalence of stunting in Riau Province (23.3%) and Indonesia (24.4%) (Kusnandar, 2022). This result identifies under-five malnutrition as the most significant factor for under-five pneumonia incidence in the Riau Province (Isnaini, 2022) and Central Java Province (Utami *et al.*, 2021). In Surabaya City, a one percent rise in children under five years of age with malnutrition increased the average of two occurrences of pneumonia under five (Maghfiroh *et al.*, 2016). It has been predicted that more than half of the under-five pneumonia mortality rates in hospitals are caused by malnutrition (Kirolos *et al.*, 2021). Based on geographical distribution, malnutrition in the Rokan Hilir District is concentrated in locations with a low percentage of exclusive breastfeeding, insufficient coverage of toddler health services, and an inadequate ratio of health facilities. To anticipate under-five malnutrition, fundamental efforts that may be performed include supporting exclusive breastfeeding, supplemental feeding, and boosting community understanding and awareness of the significance of balanced nutrition for children’s health. At a more advanced level, it is important to offer suitable under-five health facilities and services to reduce the incidence of under-five pneumonia in the Rokan Hilir District.

The proportion of healthy households plays a significant role in developing an atmosphere that promotes good health. A healthy house is a place to live that satisfies many technical health standards, so that its residents avoid various health issues and may attain optimum health status (Saragih *et al.*, 2022). There were 17 healthy home variables, including occupancy density, roof type, wall type, floor type, latrine ownership, latrine type, fecal disposal, drinking water source, distance from drinking water source to fecal storage, location of drinking water source, physical condition of water, location of hand washing, available water at the hand washing location, available hand washing liquid, lighting source, type of fuel for cooking, and location of the house (Veridona and Prabawa, 2020). Increasing the number of healthy houses may minimize the susceptibility to under-five pneumonia cases in a region (Fikri, 2016). The average percentage of healthy dwellings by subdistrict in the Rokan Hilir District was high at 71.11%, which is a counterbalancing variable to the risk of under-five pneumonia transmissions.

Although poverty levels are typically linked to inadequate access to healthcare and poor nutrition, a study in the Rokan Hilir District demonstrated that poverty does not have a clear association with an increase in under-five pneumonia cases. Similar situations also occur in patients with under-five pneumonia on Java (Renika, 2021). However, it should be highlighted that, within the conceptual framework, poor health may be a cause of poverty, and poverty can cause a low health status. This study illustrates that the government’s efforts to provide health insurance for incapacitated people through the Jaminan Kesehatan Nasional (JKN) programs, which involve BPJS Kesehatan, Kartu Indonesia Sehat (KIS), Jaminan Kesehatan Daerah (Jamkesda), and Kesehatan Ibu dan Anak (KIA), have provided equal opportunities for incapacitated people to meet their health service access needs. The implementation of JKN via BPJS Kesehatan has a significant impact on increasing access to healthcare for the incapacitated (Putri, 2014; Djunawan, 2018). Around 1.33 million persons with inadequate economic situations, or around 20.91% of the total population in Riau Province in 2020, have been enrolled as BPJS Kesehatan participants with the PBI status of Contribution Assistance Recipients (CSA Riau, 2021).

The coverage of under-five health services and the number of under-five health facilities have a major influence on the under-five pneumonia cases in the Rokan Hilir District. Adequate access to these services helps prevent and treat the condition early. Under-five healthcare coverage, which includes vaccination, growth monitoring, and dietary treatments, may successfully lower the incidence of pneumonia by boosting the immune system of under-five individuals. The Poisson regression model demonstrated that every 0.62% average increase in under-five healthcare coverage might lower the occurrence of under-five pneumonia. Similarly, a one percent increase in the number of health facilities would increase the number of under-five pneumonia cases by one. (Maghfiroh *et al.*, 2016) indicated that every one percent improvement in the coverage of under-five health services would lower the incidence of under-five pneumonia in Surabaya City. A drop in the proportion of under-five health services is assumed to be the reason for the rise in 411 under-five pneumonia cases in the Bali Province (Dewi *et al.*, 2020). Suboptimal coverage of under-five healthcare will affect the growth of under-five pneumonia cases (Guswahyuni *et al.*, 2019). A comprehensive under-five healthcare coverage, including vaccination, early diagnosis, and treatment of illness, may prevent pneumonia and its associated sequelae.

The availability of adequate health facilities offers timely access to diagnostics, case detection, treatment, and medical care, which are crucial in handling under-five pneumonia cases that frequently require urgent intervention. Low under-five healthcare coverage implies that fewer children receive critical preventative therapy, such as *Pneumococcal* and *Haemophilus influenzae type b* (Hib) vaccinations, which are beneficial for preventing pneumonia (O’brien *et al.*, 2009). The number of first-level health facilities, such as community health centers (puskesmas), has a substantial impact on the number of under-five pneumonia cases in Rokan Hilir District, where the larger the number of puskesmas, the higher the number of under-five pneumonia cases discovered. The availability of health services in a location can boost the finding of under-five pneumonia cases compared to other places with inadequate health facilities (Renika, 2021). The absence of appropriate ratios and restricted access to health treatments for children under five years of age increases the susceptibility to transmission of under-five pneumonia (Fekadu *et al.*, 2024). Therefore, boosting the coverage of under-five health services and guaranteeing the availability and proper distribution of healthcare facilities is crucial for minimizing the number of pneumonia cases in Rokan Hilir District. The gradual procurement, construction, development, and maintenance of healthcare facilities can be prioritized in the medium-term regional development plan (RPJMD) of Rokan Hilir District to achieve the ideal ratio based on population size to enhance screening and diagnostic testing services for under-five pneumonia cases.

The absence of well-coordinated nationwide programs and guidelines has hindered the prioritization of pneumonia prevention, similar to the DOTS program for TB (Marley *et al.*, 2023; Dana *et al.*, 2024). Consequently, pneumonia has not received similar importance despite it being one of the top causes of illness and death among under-fives in Indonesia (Fortina *et al.*, 2023). Without clear countrywide instructions, efforts to treat pneumonia are generally fragmented, lack coordination, and depend on regional initiatives with varying levels of effectiveness. Hence, it is essential to establish an under-five pneumonia program that encompasses a structured framework, including uniform diagnosis, supervised therapy, and vigilant monitoring, all of which play crucial roles in the effectiveness of under-five pneumonia interventions. The study found no significant correlation between the decrease in the ratio of health professionals among under-five pneumonia cases. However, improving skills and competencies through continuous education and training of health workers for effective and efficient surveillance is recommended. The equitable distribution of health workers with adequate qualifications and competencies is crucial for diagnosing, treating, and providing education about pneumonia in under-fives, which can significantly reduce morbidity and mortality rates (Okafor *et al.*, 2023).

In this study, GIS was analyzed in combination with grading of risk factors to identify areas prone to under-five pneumonia. Susceptible regions were classified into three categories: high-risk (score >14), medium-risk (score 12–14), and low-risk (score <12). This classification is based on natural break classification and Jenk optimization techniques. High-risk locations are characterized by high population density, high rainfall, high malnutrition, low health homes, exclusive breastfeeding, under-five service coverage, and several health facilities. Moderate population density, exclusive breastfeeding, healthy homes, availability of health services for children under five years of age, number of health facilities, erratic rainfall patterns, and malnutrition all have an impact on areas classified as moderate risk. Meanwhile, low-risk locations tend to have low population density, malnutrition, and rainfall; a moderate proportion of healthy homes; a high percentage of exclusive breastfeeding; under-five health service coverage; and several health facilities. A spatial study of the risk factors provides the main benefit of efficiently diagnosing and controlling the risk of pneumonia (Andrade *et al.*, 2004). By mapping the geographical distribution of pneumonia cases using an overlay of risk factors, the susceptibility of a region can be determined, allowing for more focused and efficient interventions based on priority areas. Priority regions for under-five pneumonia include the Pasir Limau Kapas, Simpang Kanan, Kubu, Bagan Sinembah Raya, and Sinaboi sub-districts. Therefore, it is necessary to reinforce interventions that target these regions through regional health planning, comprehensive response programs, and effective and efficient resource allocation.

The geographical distribution of high-risk locations surrounded by medium and low-risk areas deserves particular consideration in the under-five pneumonia response, because pneumonia is an airborne illness with rapid transmission. High-risk locations close to medium and low-risk areas have the potential to be sources of infection given population mobility and inter-regional contacts that might increase disease transmission. The spatial proximity of the local morphology may transfer pneumonia-causing organisms to new places, enhance the transmission of illness, and damage areas with lower incidence rates (Gothankar *et al.*, 2018). Therefore, a holistic and comprehensive intervention plan is required, including improvement of susceptible area-based factors. By concentrating emphasis on high-risk locations and their environs and incorporating an area-based strategy in health planning, pneumonia transmission may be repressed more effectively, lowering morbidity and death rates due to this illness. Spatial analysis helps evaluate the changes and efficacy of intervention programs over time, ensuring that resources are deployed effectively, and that reductions in under-five pneumonia cases may be accomplished sustainably (Mendes *et al.*, 2023). Thus, this area-based strategy not only enhances the public health response but also enables improved long-term planning in the under-five pneumonia response.

The implications of this study can serve as an input for the government to formulate an effective and comprehensive national strategy for tackling under-five pneumonia cases. The complexity of the under-five pneumonia risk factors outlined in this study demands multi-stakeholder collaboration, a strong political will, and adequate fiscal assistance to build a complete, systematic, and sustainable response scenario. Integrated response scenarios with detailed operational standards for under-five pneumonia cases are required to ensure that responses to risk factors are more systematic and effective. Prioritization of interventions against risk factors in vulnerable areas is necessary to achieve a national pneumonia eradication goal of less than 12 incidences per 1,000 children under five years of age in 2030.

## Conclusion

This research revealed the variables linked to the growth and regional distribution of under-five pneumonia cases. The findings revealed that population density, rainfall, exclusive breastfeeding, malnutrition, under-five service coverage, healthy houses, and the number of health facilities are variables that impact the incidence of under-five pneumonia. The number of cases of under-five pneumonia has increased with population density, rainfall, malnutrition, and several health services. At the same time, increasing percentages of exclusive breastfeeding, healthy housing, and under-five healthcare coverage may lower under-five pneumonia cases. The geographical distribution of high-risk locations for under-five pneumonia is defined by high population density, rainfall, and malnutrition levels as well as low percentages of healthy homes, exclusive breastfeeding, under-five health service coverage, and several health facilities. Recommended intervention strategies include increasing preventive and promotional health counseling, coverage of health services and facilities, housing that meets health standards, dedensification efforts through local transmigration, and spatial planning with adequate infrastructure, especially in high-risk areas. This was performed to prevent the disease from spreading to medium and low-risk zones. Preventing under-five pneumonia requires a comprehensive and integrated strategy that encompasses addressing the demographic, social, economic, health infrastructure, and environmental circumstances. Vulnerable area-based program interventions, integrated national policies, and precise operational guidelines are necessary to meet the national pneumonia incidence reduction objective of <12 incidents per 1,000 children under-five by 2030.
